# Ischemic Postconditioning Assessment in the Liver of Rats Undergoing Mesenteric Ischemia and Reperfusion

**DOI:** 10.5935/1678-9741.20160068

**Published:** 2016

**Authors:** Carlos Henrique Marques dos Santos, Ricardo Dutra Aydos, Ed Nogueira, Luciana Nakao Odashiro Miiji, Pedro Carvalho Cassino, Isadora Ishaq Alves, Nádia Meneguesso Calheiros, Milena Garcia

**Affiliations:** 1 Faculdade de Medicina da Universidade Federal de Mato Grosso do Sul, Campo Grande, MS, Brazil.

**Keywords:** Mesenteric Vascular Occlusion, Ischemia, Ischemic Postconditioning, Rats, Wistar, Reperfusion Injury

## Abstract

**Introduction:**

Ischemic postconditioning is a method that shows evidence of efficacy in minimizing reperfusion injury; however, its effectiveness in preventing injuries in distant organs is still unknown, especially in those who have undergone mesenteric ischemia and reperfusion.

**Objective:**

To evaluate the effect of ischemic postconditioning in preventing reperfusion injury in the liver of rats submitted to mesenteric ischemia and reperfusion, comparing two different methods of ischemic postconditioning.

**Methods:**

30 Wistar male rats were used, distributed into three groups: Group A: Ten rats submitted to intestinal ischemia for 30 minutes followed by reperfusion for 60 minutes; Group B: Ten rats subjected to ischemia and reperfusion; after ischemia, two cycles of reperfusion (two minutes each) interleaved with two cycles of ischemia (two minutes each); and Group C: Ten rats subjected to ischemia and reperfusion; after ischemia, four cycles of reperfusion (30 seconds each) interspersed with four cycles of ischemia (30 seconds each). After the experiment, the left lobe of the liver was resected for subsequent histological analysis, using the following classification: grade 1 - centrilobular congestion; grade 2 - centrilobular congestion with some degeneration of hepatocytes in one or two central veins; and grade 3 - multifocal centrilobular congestion and degeneration of portal hepatocytes.

**Results:**

The mean degree of liver damage found was 1.8 in group A, 1.7 in group B and 1.3 in group C. There was no statistically significant difference between the groups.

**Conclusion:**

Ischemic postconditioning was unable to minimize reperfusion injury in rats undergoing mesenteric ischemia and reperfusion.

**Table:** 

Abbreviations, acronyms & symbols	
IPC =	Ischemic postconditioning
I/R =	Ischemia and reperfusion

## INTRODUCTION

Ischemia, regardless of the affected organ, is an important cause of mortality in our country. Reperfusion, although essential, is considered a factor of clinical deterioration of the patient due to the formation of toxic reactive oxygen species, promoting cell injury, bacterial translocation and systemic inflammatory response, with no effective treatment at this time^[1,2]^.

Although intestinal ischemia accounts for only 800 in 100,000 admissions^[3]^, it has high mortality rates, ranging from 60% to 100%^[4]^, due to local lesions and distance, and it may occur as a result of multiple organ dysfunction. In this case, the liver is a major organ involved, thus its study in this process becomes relevant^[5]^.

Aiming to address the various situations of ischemia avoiding reperfusion lesions, a large number of substances and procedures have been studied, including its remote and local effects. Some of the published proposals obtained good experimental results, but without proven success in clinical practice^[6,7]^.

In 2003, Zhao et al.^[8]^ proposed an alternative treatment of ischemia and reperfusion (I/R), ischemic postconditioning (IPC), which consists of performing one or more cycles of reperfusion followed by one or more cycles of ischemia, before the reperfusion phase, demonstrating a protective effect on myocardial ischemia in animals.

In mesenteric I/R, IPC was initially assessed by Santos et al.^[9]^, who also observed its effectiveness in this process, which was subsequently verified and published by other authors. However, there are no studies assessing IPC's ability to reduce liver damage in mesenteric I/R, making it necessary to carry out further studies to define its role in this condition.

The aim of this study is to evaluate the effect of IPC in preventing reperfusion injury in the liver of rats subjected to mesenteric I/R by comparing two different methods of IPC.

## METHODS

The study was approved by the Ethics Committee of the Federal University of Mato Grosso do Sul. All ethical rules established by the Brazilian College of Animal Experimentation were followed.

Thirty rats (*Rattus norvegicus albinos,* Rodentia, Mammalia), Wistar male adult, were obtained from the vivarium of the Federal University of Mato Grosso do Sul and divided into three groups:

- Group A - I/R: comprised of ten rats subjected to intestinal ischemia by occlusion of the cranial mesenteric artery with a vascular clamp for 30 minutes, followed by reperfusion for 60 minutes.
- Group B - IPC 1: comprised of ten rats subjected to ischemia by occlusion of the cranial mesenteric artery with a vascular clamp for 30 minutes and reperfusion for 60 minutes. Between ischemia and reperfusion, two reperfusion cycles (two minutes each) interleaved with two ischemia cycles (two minutes each) were performed.
- Group C - IPC 2: comprised of ten rats subjected to ischemia by occlusion of the cranial mesenteric artery with a vascular clamp for 30 minutes and reperfusion for 60 minutes. Between ischemia and reperfusion, four reperfusion cycles (30 seconds each) interspersed with four cycles of ischemia (30 seconds each) were performed.

The animals were weighed on an electronic scale and anesthetized by intraperitoneal injection of 2:1 solution of 50 mg/mL ketamine hydrochloride (Cetamin^®^), and 20 mg/mL xylazine (Xilazin^®^), at a dose of 0.1 mL/100g.

The rats were maintained under spontaneous ventilation throughout the procedure. Median longitudinal laparotomy of about four centimeters, externalization of the small intestine, as well as identification and dissection of the cranial mesenteric artery were performed.

In group A, the cranial mesenteric artery was occluded by atraumatic vascular clamp for 30 minutes (ischemia phase). After placing the clamp, the small intestine was repositioned in the abdominal cavity and the wound was closed with continuous suture of the skin with nylon monofilament 4-0 (mononylon^®^). After the ischemia phase, the abdominal wall was opened again by removing the suture and the vascular clamp was removed, beginning the reperfusion phase, which lasted 60 minutes. In all three groups, reperfusion was initiated, the abdomen was closed once again by continuous suture of the skin with nylon monofilament 4-0 until the end of the experiment.

In group B, 30 minutes of ischemia and 60 minutes of reperfusion were carried out. Preceding the reperfusion, IPC was performed, by carrying out two reperfusion cycles (removal of atraumatic vascular clamp of the cranial mesenteric artery), lasting two minutes each, interspersed with two ischemia cycles (occlusion of the cranial mesenteric artery by atraumatic vascular clamp), also lasting two minutes each.

**Fig. 1:**
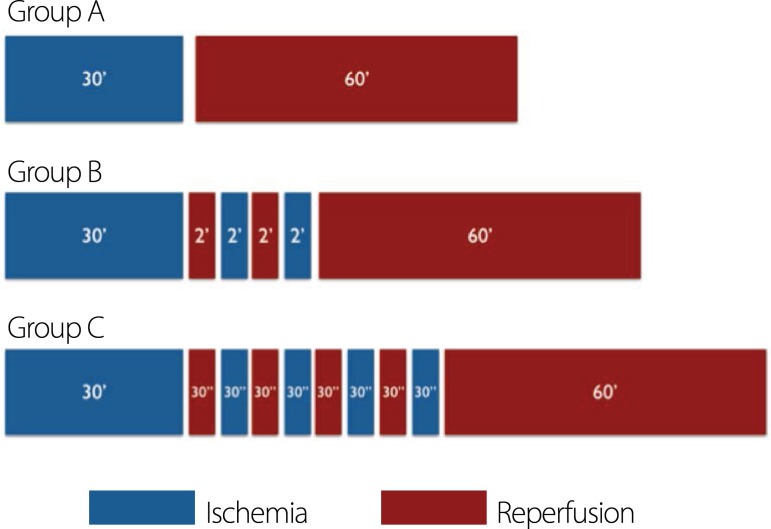
Schematic demonstration of time used for ischemia and reperfusion in group settings.

In group C, 30 minutes of ischemia and 60 minutes of reperfusion were carried out. Preceding the reperfusion, IPC was performed, by carrying out four reperfusion cycles (removal of atraumatic vascular clamp of the cranial mesenteric artery), lasting 30 seconds each, interspersed with four ischemia cycles (occlusion of the cranial mesenteric artery by atraumatic vascular clamp), also lasting 30 seconds each (Figure 1).

After reperfusion, in the three groups, the abdominal wall was opened again by removing the suture and the left lobe of the liver was resected, washed with saline and placed in a 10% solution of formaldehyde for subsequent histological analysis. The animals were euthanized by increasing the anesthesia level.

After fixation in 10% formaldehyde solution, the resected liver segments were submitted to histological processing. The slides were stained with hematoxylin-eosin and analyzed in optical microscope by a pathologist without prior knowledge of the group each rat belonged to, who then ranked them according to the degree of tissue injury. To this end, the following rating was used, according to prior publication of Takeda et al.^[10]^: Grade 0 - no histological changes; Grade 1 - centrilobular congestion; grade 2 - centrilobular congestion with some degeneration of hepatocytes in one or two central veins; and grade 3 -multifocal centrilobular congestion and degeneration of portal hepatocytes.

The results were analyzed statistically using ANOVA variance test, and were considered significant if *P*<0.05.

## RESULTS

Macro and microvesicular steatosis were identified in some cases, mostly close to the terminal hepatic veins. Groups A and B showed degrees of injury between 1 and 3, corresponding to 1.8 and 1.7, respectively, whereas degrees of injury obtained in Group 3 were 1 and 2, with a mean of 1.3 (Table 1). There was no statistical difference between the groups: *P*=0.748 between groups A and B, *P*=0.068 between groups A and C, and *P*=0.127 between groups B and C.

**Table 1:** Results of the degree of liver damage observed in animals, according to the groups.

Rats	Group A (I/R)	Group B (IPC 1)	Group C (IPC 2)
1	1	1	1
2	2	1	2
3	1	2	1
4	1	3	1
5	2	2	2
6	3	1	1
7	1	1	2
8	1	1	1
9	3	3	1
10	3	2	1
Average	1.8	1.7	1.3

*P*=0.748 between groups A and B; *P*=0.068 between groups A and C; *P*=0.127 between groups B and C

## DISCUSSION

It is known that the consequences of ischemia in different tissues, depending on its duration, and many of the resulting injuries are developed during the tissue reoxygenation stage. One of the organic mechanisms of greater impact for cell homeostasis is oxidative stress and the production of free radicals, resulting from two major pathophysiological events: ischemia followed by reperfusion and the inflammatory process culminating with local and/or systemic changes^[11]^.

Mesenteric ischemia is one of the most serious diseases of the gastrointestinal tract and, depending on its development time, the process can evolve to necrosis when blood flow is restored, aggravating the damage occurred in the ischemic phase. Injury to the intestinal mucosa from I/R is well known, but little is known about the involvement of the digestive tract portions, the focus distance of the primary lesion, even though remote injury has been extensively documented in other situations of I/R^[12,13]^.

Seifi et al.^[14]^ evaluated the protective effect of IPC on liver damage after kidney I/R. Rats underwent renal ischemia for 45 minutes and IPC was carried out in four cycles of I/R, each with a 10-second duration. It was observed that the kidney I/R caused a significant increase in liver function indices, such as increased transaminases. On the other hand, those parameters were significantly reduced in the IPC group, showing induced reduction of malondialdehyde levels in the liver and increased superoxide dismutase activity. In this study, although the organ subjected to I/R was the liver, carrying out a larger number of shorter cycles in the same IPC was not more efficient than performing a smaller number of cycles of longer duration.

In the evaluation of IPC in aortic I/R of rats, Dorsa et al.^[15]^ also studied its effectiveness in protecting a distant organ, showing less damage to the lung parenchyma in this group compared to the control group. The authors performed aortic clamping for 30 minutes and reperfusion for 60 minutes, with three cycles of IPC lasting two minutes each. In this study, IPC performed with the same time span used by Dorsa et al.^[15]^ did not result in hepatic protection, obtaining results similar to the I/R group. However, there is still a lot of controversy in the literature on the outcome of remote protection of IPC. Recently, Santos et al.^[16]^ also evaluated the possible protective action of IPC on lung parenchyma when performing mesenteric I/R in rats and found no benefits in using this technique. Similarly, the research presented here showed no liver protection with IPC on mesenteric I/R, leaving some possibilities to be considered, still with no clear answers. First, the times employed in IPC and the number of cycles can influence the result. This would be true when comparing these two studies evaluating IPC pulmonary action, since there was a difference in the method and this has been the greatest difficulty when comparing the numerous studies of IPC - the wide variation in the method used. The second question refers to the origin of ischemia: Dorsa et al.^[15]^ performed aortic clamping while Santos et al.^[16]^ and the present study used mesenteric ischemia. This factor may have some influence since in the aortic clamping, the harmful products of the I/R would spread to all the organs and tissues, whereas in the intestinal I/R, all blood is primarily drained to the liver, thus leading to increased toxic concentration of reactive oxygen species on this organ. Thirdly, one should consider that there may be some differences in resistance to reperfusion injury; therefore, a valid protection mechanism for the lung tissue cannot display the same efficacy in the liver parenchyma.

The effectiveness of IPC in minimizing liver reperfusion injury had already been demonstrated by Santos et al.^[17]^, through the evaluation of its effect directly on liver I/R, as opposed to this study, which analyzed its remote effect. That study showed the effectiveness of the method with three cycles of IPC lasting 30 seconds each, which corroborates the findings of this research, which also found better results with shorter cycles, although there was no statistically significant difference.

The literature shows evidence that there may be possible differences in response between the various organs studied for IPC. This technique has already been proven as effective in intestinal I/R protection^[9]^, nevertheless, Nakamura et al.^[18]^ recently demonstrated that five cycles with a duration of thirty seconds each for IPC were not able to prevent reperfusion injury in rats. Failure to protect tissue through IPC using short cycles had already been published previously by Bretz et al.^[19]^, who performed jejunal I/R in rabbits. The authors used IPC for four cycles of 30 seconds each, as used here, without showing advantages over the control group. It is important to note, however, that both aforementioned studies only made the assessment in tissue subjected to the I/R process and not in distant organs such as the present research.

Although several publications have demonstrated the effectiveness of IPC in different situations of I/R since its original publication, there are still doubts about its best application in terms of the number of cycles and their duration, especially when considering their action at a distance. This research has shown that, in intestinal I/R in rats, there was no difference between the two methods of IPC applied and that they were unable to minimize reperfusion injury. Further studies should be performed in order to conclude how it could be more effective for remote I/R.

## CONCLUSION

IPC was unable to minimize reperfusion injury in rats undergoing mesenteric ischemia and reperfusion.

**Table:** 

Authors’ roles & responsibilities	
CHMS	Execution of operations and/or trials; analysis and/or data interpretation; manuscript writing or critical review of its content; final manuscript approval
RDA	Final manuscript approval
ENN	Execution of operations and/or trials; final manuscript approval
LNOM	Analysis and/or data interpretation; final manuscript approval
PCC	Execution of operations and/or trials; final manuscript approval
IIA	Statistical analysis; final manuscript approval
NMC	Manuscript writing or critical review of its content; final manuscript approval
MG	Execution of operations and/or trials; final manuscript approval
